# Exploring the Potential of a Genome-Reduced *Escherichia coli* Strain for Plasmid DNA Production

**DOI:** 10.3390/ijms241411749

**Published:** 2023-07-21

**Authors:** Thi Thuy Nguyen, Le Minh Bui, Ji-Young Byun, Byung-Kwan Cho, Sun Chang Kim

**Affiliations:** 1 Department of Biological Sciences, Korea Advanced Institute of Science and Technology (KAIST), Daejeon 34141, Republic of Korea; 2 Department of Biotechnology, NTT Hi-Tech Institute, Nguyen Tat Thanh University (NTTU), Ho Chi Minh City 700000, Vietnam; 3 KI for BioCentury, Korea Advanced Institute of Science and Technology (KAIST), Daejeon 34141, Republic of Korea

**Keywords:** *Escherichia coli*, minimal genome, genome reduction, plasmid DNA, antibiotics, antibiotic sensitivity

## Abstract

The global demand for nucleic acid-based vaccines, including plasmid DNA (pDNA) and mRNA vaccines, needs efficient production platforms. However, conventional hosts for plasmid production have encountered challenges related to sequence integrity due to the presence of insertion sequences (ISs). In this study, we explored the potential of a genome-reduced *Escherichia coli* as a host for pDNA production. This strain had been constructed by removing approximately 23% of the genome which were unessential genes, including the genomic unstable elements. Moreover, the strain exhibits an elevated level of NADPH, a coenzyme known to increase plasmid production according to a mathematical model. We hypothesized that the combination of genome reduction and the abundance of NADPH would significantly enhance pDNA production capabilities. Remarkably, our results confirmed a three-fold increase in pDNA production compared to the widely employed DH5α strain. Furthermore, the genome-reduced strain exhibited heightened sensitivity to various antibiotics, bolstering its potential for large scale industrial pDNA production. These findings suggest the genome-reduced *E. coli* as an exciting candidate for revolutionizing the pDNA industry, offering unprecedented efficiency and productivity.

## 1. Introduction

Nucleic acid vaccines have become a favorable alternative to conventional vaccines because of their high capability for rapid development and potential for low-cost manufacture and safe administration [1,2]. mRNA vaccines require a hundred micrograms, whereas milligram amounts of pure, supercoiled pDNA are needed to initiate immune response [3,4]. Consequently, both of these nucleic acid-based vaccines, plasmid DNA (pDNA) and mRNA, require an industrial scale production of pDNA. 

Unfortunately, the COVID-19 outbreak posed additional difficulties in the production of pDNA due to the high demand. The biopharmaceutical industry has been operating with exceptional speed to produce vaccines that are safe and effective against SARS-CoV-2 coronavirus. With the combined requirements of the nucleic acid vaccines and cell and gene therapy industries, DNA manufacture has reached a point where demand far exceeds the available capacity [5,6,7]. Therefore, pDNA production has emerged as a critical bottleneck in the genetic medicine revolution, requiring DNA manufacturers to respond quickly and effectively [7].

In order to meet strict compliance and regulatory standards in biopharmaceutical applications, it is crucial to attain not only a high pDNA yield but also excellent quality. There has been a major concern about sequence integrity when using conventional hosts for pDNA production [8,9,10]. pDNA is conventionally produced in *E. coli* strains such as DH5α, DH10B and JM109 [11,12] because of the *endA* and *recA* negative phenotype, which prevents plasmid degradation and nicking during cell lysis [13,14,15]. However, depending on the specific strains and/or plasmids involved, the effects of these selective mutations can vary significantly [14]. These conventional *E. coli* strains harbor hundreds of insertion sequences (ISs) within their genomes, which have the potential to induce various genetic changes, including inversions, deletions, duplications, and the facilitation of horizontal gene transfer. For instance, DH10B exhibits a higher mutation rate—even higher than wildtype strain MG1655—due to an increased rate of insertion sequence (IS) transposition [16]. In industrial applications, IS transposition has been observed to cause low plasmid production during the selection of HIV DNA vaccine candidates, and instability in plasmids carrying engineered genes, resulting in a significant decline in protein production efficiency [8,17,18]. The presence of mobile elements in plasmid DNA has raised regulatory concerns due to their potential to modify the biological characteristics and safety attributes of the vector DNA. Consequently, it is crucial to employ well-characterized strains that can minimize the pDNA sequence instability caused by IS to ensure the safe and reliable production of pDNA in industrial applications.

Genome-reduced *Escherichia coli* strains, obtained through targeted deletions of non-essential genomic regions, offer a solution to these challenges. These strains have been constructed from the wildtype K-12 strain by eliminating mobile genetic elements such as insertion sequences (ISs), transposases, phages, integrases, and recombinases [19], and further deleting nonessential genes including K-islands, fimbriae, flagella, part of the LPS synthetic genes, and genes responsible for anaerobic respiration [20]. The genome-reduced strain, MSD42, has been proven to enhance the stability of lentiviral vector [21] and MS56 was demonstrated to increase the production of recombinant proteins [20]. However, the evaluation of pDNA yield from these genome-reduced strains has not been conducted.

Even though genome-reduced *E. coli* strains offer clear advantages, their application on an industrial scale has been limited so far. One possible reason is that these strains often show impaired growth under laboratory conditions, particularly in minimal medium [22]. Therefore, Choe et al. [22] evolved the MS56 strain [20] to recover its growth in minimal medium and then generated the eMS57 strain. In eMS57, the NADPH level was 4.5-fold higher than the wild-type MG1655, which offers a distinct advantage for improving the pDNA production, as it has been shown through a mathematical model that increasing the availability of NADPH has a positive influence on pDNA production [23]. However, evaluation of this genome-reduced strain in plasmid DNA production has not yet been reported, but the advantages of using eMS57 are clear, and they have great potential for higher production of pDNA. Therefore, in this study, we investigated the suitability of the eMS57 strain as a host for plasmid production.

## 2. Results and Discussion

### 2.1. Re-Introduction of mutS Gene into eMS57 Strain

**Figure 1:**
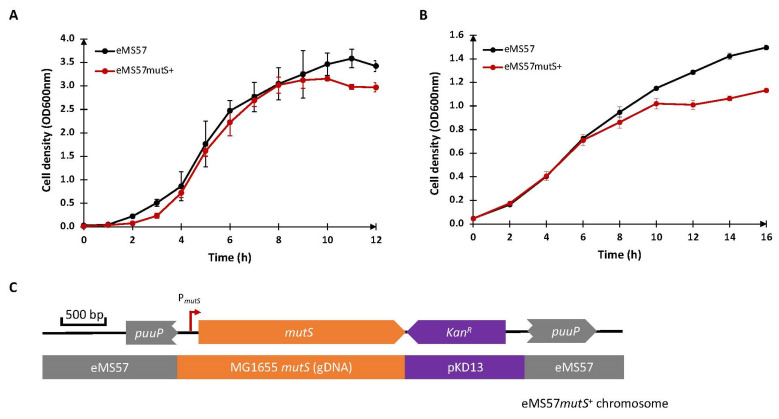
Growth profile of eMS57 and eMS57*mutS**^+^*****.** (**A**) Growth profile of eMS57 (black line) and eMS57*mutS^+^* (red line) in LB medium. (**B**) Growth profile of eMS57 (black line) and eMS57*mutS^+^*(red line) in M9G medium. (**C**) The integration site of *mutS* knocked into the genome of the eMS57*mutS^+^* strain. Figure 1C was modified from Choe et al. [22]. Error bars represent standard error of the mean.

In a previous study [22], an adaptive evolution experiment resulted in a spontaneous large deletion in eMS57, spanning 21 Kb including the *mutS* gene, a crucial component of the MutHLS DNA repair system. However, the absence of this gene compromised genome stability. To address this issue, an attempt had been made to reintroduce the *mutS* gene into the eMS57 genome using the Tn5 transposon, resulting in the creation of the eMS57*mutS^+^* strain [22]. Unfortunately, the strain retained the kanamycin resistance marker in the genome, reducing the plasmid options with antibiotic resistance that can be used with this strain. Additionally, although the strain showed similar growth to parental strain eMS57 in lysogeny broth (LB) medium (Figure 1A), it displayed poor growth, particularly in M9 glucose (M9G) medium (Figure 1B). The cause of this phenomenon is currently unknown. We propose that the insertion of the *mutS* expression cassette (Figure 1C) into *puuP*, one of the putrescine symporter genes, has disrupted the function of PuuP protein, which is highly active during the exponential growth phase in M9-tryptone medium [24]. Consequently, the disruption of the *mutS* cassette in this strain could lead to reduced uptake of putrescine during exponential growth, resulting in the slow growth observed in M9G medium. Therefore, it is necessary to generate a new strain that restores the presence of *mutS* without the aforementioned limitations. 

**Figure 2:**
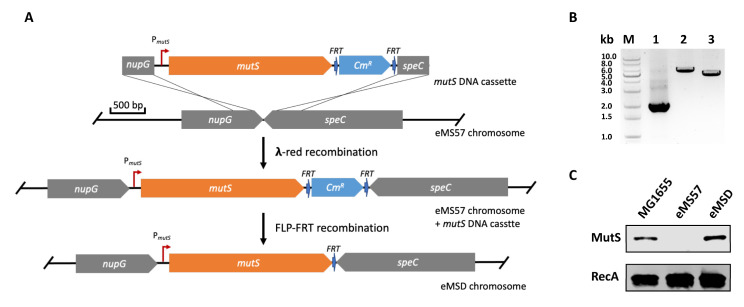
Re-introduction of *mutS* gene into the genome of the eMS57 strain. (**A**) A scheme representing the chromosome integration of the native *mutS* into the locus between *nupG* and *speC* in eMS57 using λ-red recombination, followed by the elimination of the chloramphenicol resistance marker through FLP–FRT recombination. (**B**) PCR analysis using nupG_out_F and speC_out_R primers confirms the integration of the *mutS* DNA cassette and the removal of the selection marker. Lane 1, nupG-speC fragment in the eMS57 strain; lane 2, nupG-mutS DNA cassette-speC fragment in the eMS57 *mutS* DNA cassette strain; lane 3, nupG-mutS-speC fragment in the eMSD strain; M: DNA ladder. (**C**) Western blot analysis confirms the expression of MutS protein in the MG1655, eMS57 and eMSD strains with RecA production as a control.

In this study, we utilized the λ-red homologous recombination method [25,26] to re-introduce the *mutS* gene to the genome of the eMS57 strain. The integration site chosen was between the *nupG* and *speC* genes, as this location has been previously reported as an effective expression site in the *E. coli* genome [27,28]. The integration process is illustrated in Figure 2A, and further details can be found in the Methods section. The successful integration of *mutS* into the eMS57 genome at the targeted locus was confirmed by genomic PCR (Figure 2B), and the expression of *mutS* was validated using Western blot analysis (Figure 2C). The generated strain is referred as eMSD, “eMS” is derived from the parental strain, eMS57, and the letter “D” represent the strain’s purpose for plasmid DNA production. 

**Figure 3:**
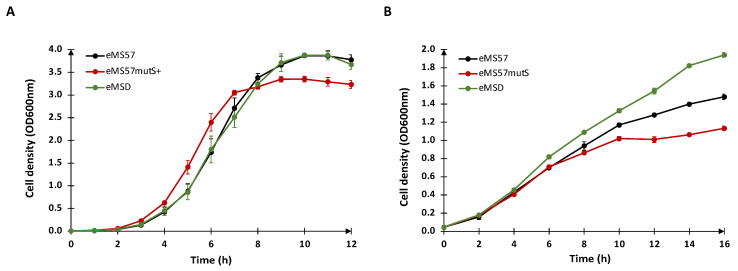
Growth profile of eMSD. (**A**) Growth profile of eMSD (green line), eMS57 (black line) and eMS57*mutS^+^* (red line) in LB medium. (**B**) Growth profile of eMSD (green line), eMS57 (black line) and eMS57*mutS^+^*(red line) in M9G medium.

Next, we evaluated the growth profile of the newly engineered strain, eMSD, in comparison to the eMS57*mutS^+^* strain and the parental strain, eMS57. The growth analysis was performed in both LB (Figure 3A) and M9G (Figure 3B). The results demonstrate that the eMSD strain exhibits a growth profile similar to the eMS57 strain in LB medium and even shows improved growth in M9G. 

### 2.2. Evaluation of Plasmid DNA Production in eMSD Strain

**Figure 4:**
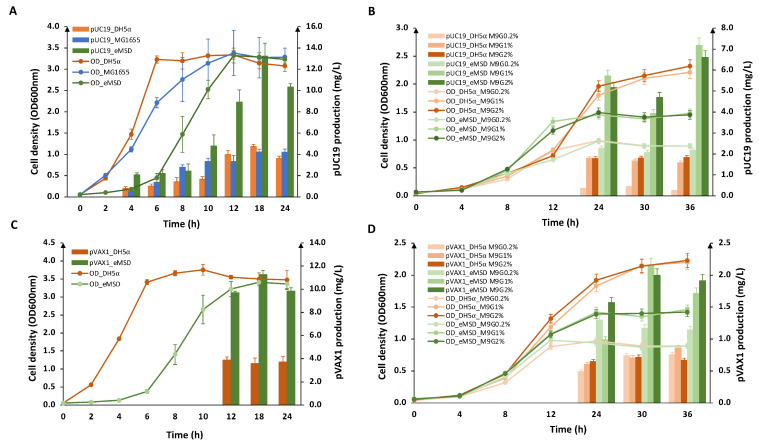
Plasmid production examined in eMSD in comparison with the DH5α strain at a 10 mL culture scale. (**A**) Growth profile and pUC19 productivity of the eMSD strain (green), DH5α strain (orange), and MG1655 (blue) in LB medium. (**B**) Growth profile and pUC19 productivity of eMSD strain (green) and DH5α strain (orange) in M9 medium with 0.2%, 1% and 2% glucose. (**C**) Growth profile and pVAX1 productivity of the eMSD strain (green) and DH5α strain (orange) in LB medium. (**D**) Growth profile and pVAX1 productivity of the eMSD strain (green) and DH5α strain (orange) in M9 medium with 0.2%, 1% and 2% glucose.

Initially, we compared the pDNA production capability of the newly generated strain eMSD with that of the DH5α strain and the wild-type strain, MG1655. We conducted cell culture in 10 mL medium to produce pUC19 (2686 bp) using LB medium and observed the highest pDNA yield after 18 h of culture (Figure 4A). This finding is consistent with previous studies that have shown the highest plasmid yields are typically obtained during the stationary phase [12,14,29]. The results also showed that eMSD outperformed both DH5α and MG1655 strains in pUC19 production. eMSD produced approximately three-fold higher pUC19 compared to the DH5α and MG1655 strains, while maintaining similar cell density. This significant increase in plasmid production in the eMSD strain is likely due to the combined effects of genome reduction and the abundance of NADPH that has been proven to enhance pDNA production through a mathematical model described in a previous report [23].

As the DH5α strain is widely used as a benchmark strain for comparing plasmid production capabilities, subsequently, we examined the plasmid production of eMSD and compared it to this strain. Figure 4B shows the pUC19 production of these two strains in M9 medium supplemented with different concentrations of glucose (0.2%, 1.0%, and 2.0%). In minimal medium with glucose supplementation, the density of DH5α cells increased significantly (~2.5 fold) with increasing glucose concentration. On the other hand, the eMSD strain showed a smaller increase (~1.7 fold) in the cell density in the culture supplemented with 1% glucose, compared with that of the culture containing 0.2% glucose. There is no significant difference between the cell density of the two strains in M9G1% and M9G2% cultures. In terms of pUC19 production in minimal medium, both strains produce fewer plasmids than LB medium. The eMSD strain produced up to 8.4, 4.5, and 3.6-fold more pUC19 than DH5α in M9G0.2%, M9G1%, and M9G2%, respectively, after 36 h of culture. With higher glucose supplementation (1% and 2%), the pUC19 production increased 3.3-fold compared to low glucose supplementation while there is not much change in plasmid production in DH5α. There was no significant difference in pUC19 production between 1% and 2% glucose supplementation for both strains. In the same conditions, it appears that eMSD utilized resources more efficiently for pDNA production, rather than increasing biomass like the DH5α strain. These results support our hypothesis that the eMSD strain is much more efficient in pDNA production compared to the conventional DH5α strain.

Subsequently, we performed similar experiments to produce pVAX1 (2999 bp), a backbone vector for pDNA vaccines (Figure 4C,D). We obtained similar results, although the productivity of pVAX1 in minimal media was lower than that of pUC19 in both strains, and the differences between the three minimal media were less pronounced. It has been previously observed that different plasmids can result in different productivities within the same strains [14]. Nevertheless, eMSD demonstrated more efficient production of pVAX1 compared to DH5α.

**Figure 5:**
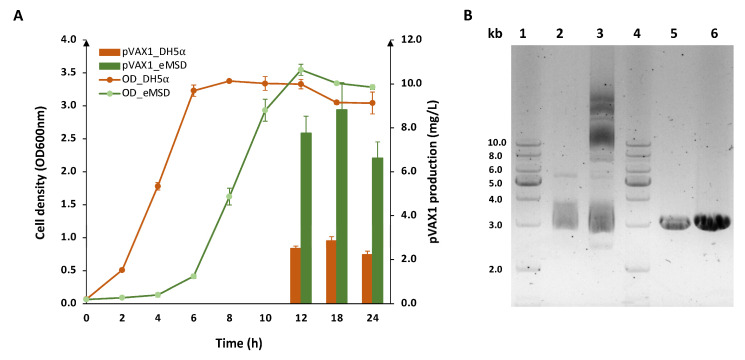
pVAX1 production from the eMSD strain compared to the DH5α strain in 1 L LB medium. (**A**) Growth profiles and pVAX production of the eMSD strain (green) and DH5α strain (orange) in 1 L LB medium with flask culture are shown. (**B**) Gel electrophoresis analysis of purified pDNA from both strains after 18 h of culture on a 1% agarose gel. Lane 1 and 4, DNA ladder; lane 2, pVAX1 from the DH5α strain; land 3, pVAX1 from the eMSD strain; lane 5, pVAX1 from DH5α digested with EcoRI enzyme; lane 6, pVAX1 from eMSD digested with EcoRI enzyme.

In addition, we scaled up the production of pVAX1 in 1 L LB medium using 3 L flasks and compared it with DH5α to assess whether the results were consistent at a larger scale. As expected, eMSD produced up to a 3.1-fold higher titer of pVAX1 than that of the conventional strain after 18 h culture (Figure 5A). The undigested and digested pVAX1 purified from both strains after 18 h of culture were analyzed on a 1% agarose gel (Figure 5B). In terms of productivity, the result once again confirmed the enhanced productivity of eMSD in consistently producing pDNA at different culture scales. Particularly, the undigested product from the eMSD strain exhibited a distinct supercoiled band, distinguishing it from DH5α. Despite not being specifically engineered for pDNA production, our strain has already surpassed the conventional strain in terms of pDNA productivity and it shows great potential for higher quality production. 

### 2.3. The Antibiotic Sensitivity of the eMSD Strain

The high sensitivity of a biopharmaceutical platform to various antibiotics can be advantageous in industry. It allows for precise control over the growth and survival of the host strain during the production process. By using antibiotics as selective agents, it becomes easier to maintain the purity and integrity of the production strain, minimizing the risk of contamination by unwanted bacteria. Furthermore, antibiotics help remove the strain after production, resulting in a safe and efficient process. Last but not least, it can contribute to reducing costs during the production process. Therefore, it is necessary to examine the sensitivity of our strain to various common antibiotics. 

**Figure 6:**
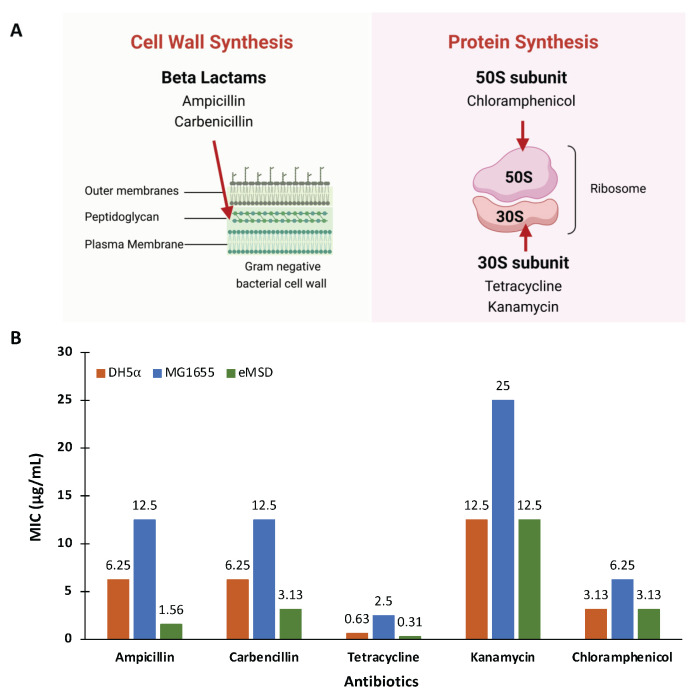
Minimum inhibitory concentrations (MIC) of various antimicrobials against the eMSD strain. (**A**) Mechanisms of action of tested antibiotics. Ampicillin and carbenicillin inhibit peptidoglycan synthesis, which is crucial for cell wall information. Tetracycline and kanamycin target the 30S subunit of ribosome while chloramphenicol specifically targets the 50S subunit involved in protein synthesis. The red arrows indicate the respective targets of these antibiotics. (**B**) The MIC values of ampicillin, carbenicillin, tetracycline, kanamycin and chloramphenicol were measured against the eMSD strain (green bar) in comparison to the wild type strain MG1655 (blue bar) and DH5α strain (orange bar).

We conducted sensitivity tests on our strain using five commonly used antibiotics with different mechanisms of action (Figure 6A) [30]. These antibiotics included β-lactams (ampicillin and carbenicillin), which target peptidoglycan in cell wall synthesis, tetracycline and kanamycin, which target the 30S subunit, and chloramphenicol, which targets the 50S subunit of ribosome involved in protein biosynthesis. We compared the results with those of the wildtype strain MG1655 and the DH5α strain. As shown in Figure 6B, the eMSD strain is more sensitive to all five tested antibiotics compared to the MG1655 strain. It is noteworthy that no antibiotic-resistance genes were removed during the strain generation process. The specific genes that were deleted from the MG1655 strain can be found in previous reports [20,22]. Additionally, eMSD showed increased sensitivity to ampicillin, carbenicillin and tetracycline compared to the DH5α strain. We hypothesize that the changes in membrane permeability in our strain, resulting from the genome reduction, contribute to its increased sensitivity to antibiotics. This hypothesis is also supported by a previous study [31]. This characteristic provides more advantages in our strain as a platform for plasmid production.

## 3. Materials and Methods

### 3.1. Strains, Media, Primers, Enzymes, Chemicals, and Instruments

The bacterial strains used in this study are described in Table 1. XL1-Blue was used for all cloning experiments. All primers listed in Table 2 were synthesized at Genotech (Daejeon, South Korea). M9 glucose medium (M9G) contains 47.75 mM of Na_2_HPO_4_, 22.04 mM of KH_2_PO_4_, 8.56 mM of NaCl, 18.7 mM of NH_4_Cl, 2 mM of MgSO_4_, 0.1 mM of CaCl_2,_ and 20 g/L of glucose, unless specified otherwise. For culturing DH5α in M9G medium, 0.6 g/L thiamine was added. Terrific Broth (TB) medium contains 24 g/L of yeast extract, 20 g/L of tryptone, 4 mL/L of glycerol, 0.17 M of KH_2_PO_4_, and 0.072 M of K_2_HPO_4_. Lysogeny broth (LB) medium was prepared by dissolving 20 g LB broth low salt (Duchefa Biochemie, Haarlem, The Netherlands) in 1 L of demineralized water. All restriction enzymes were from New England Biolabs. Unless otherwise stated, all chemicals were from Sigma-Aldrich (St. Louis, MO, USA). Optical density at 600 nM (OD_600_) was measured by a photometer Genesys 20 (Thermo Scientific, Waltham, MA, USA).

### 3.2. Construction of mutS DNA Cassette

To construct a DNA cassette for integrating *mutS*, we first amplified the *nupG* (500 bp upstream) and *speC* (500 bp downstream) fragments as homologous arms from the eMS57 genome with primers XbanupGF and nupGXENNR and oopspeCF and SpeCNdeR, respectively. The *nupG* fragment was then cloned into the pUC19 vector at the XbaI and NdeI restriction sites. Next, we amplified from pACYCDuet-1 vector the *Cm^R^* fragment 1 with the FRT sequence upstream using primers EcoRIFRTCmR1F and CmR1NcoR. This DNA fragment was cloned into the pUC19-*nupG* vector at the EcoRI and NcoI sites as pUC19-*nupG*-FRT-CmR1. The *Cm^R^* fragment 2 with the 2nd FRT sequence downstream was amplified from the pACYCDuet-1 vector with primers CmR2NcoF and CmR2FRTR. The *Cm^R^* fragment 2 and *speC* fragment were assembled by overlapping PCR to create the CmR2-*speC* fragment. This fragment was then cloned into pUC19-*nupG*-FRT-CmR1 to create the pUC19-*nupG*-FRT-*Cm^R^*-FRT-speC vector. Finally, we cloned the native *mutS* fragment amplified from the genomic DNA of MG1655 using XhoI_mutS_F and mutS_EcoRI_R into pUC19-*nupG*-FRT-*Cm^R^*-FRT-*speC* at XhoI and EcoRI sites to complete a vector containing a *mutS* DNA cassette, referred as pUC19-*nupG*-*mutS*-FRT-*Cm^R^*-FRT-*speC*. The successfully constructed plasmids were confirmed by DNA sequencing. This vector was digested with EcoO109I and HindIII before being used as the template for a PCR reaction to amplify the *mutS* DNA cassette. All the primer sequences are listed in Table 2.

### 3.3. Genome Integration of mutS Gene

To integrate the *mutS* DNA cassette into the eMS57 genome, we first prepared electro-competent cells of eMS57 containing the pREDI vector following the method described by Yu et al. [26] with some modifications, using TB medium instead of LB medium. The cells were harvested at an OD_600_ of 1 during the early log phase in TB medium. A total of 1 μg DNA cassette was electro-transformed into 100 μL of the electro-competent cells. The transformed cells were inoculated in 10 mL of TB medium at 30 °C for 3 h. After incubation, the cells were harvested and spread onto TB plates containing ampicillin and chloramphenicol and incubated at 30 °C until colonies appeared. To confirm the presence of the *mutS* DNA cassette in the genome, genomic DNA PCR was performed using nupG_out_F and speC_out_R primers. To remove the selection marker, the correct clone was transformed with the pCP22 vector after eliminating the pREDI vector. Flippase (FLP) recombinase, expressed from the pCP22 vector, excised the marker cassette. The correct candidates were then screened by genomic PCR using nupG_out_F and speC_out_R primers to confirm the removal of the *Cm^R^* gene.

### 3.4. Genome and Plasmid Purification

Genomic DNA was extracted from 1 mL of overnight culture using Exgene^TM^ Cell SV mini (GeneAll, #106-101), following the instructions of the manufacturer and eluting the genomic DNA in 100 μL of TE buffer. Plasmid DNA was purified from 1 mL of culture using the Qiaprep Spin Miniprep kit (Hilden, Germany), following the instructions of the manufacturer and eluting the plasmid DNA in 50 μL of TE buffer. DNA concentration was measured spectrophotometrically at 260 nm using a Nanodrop UV spectrophotometer (NanoDrop 2000, Thermo Scientific, Waltham, MA, USA). 

### 3.5. Western Blot Analysis

60 μL of total protein from cell lysate was separated by sodium dodecyl sulfate polyacrylamide gel electrophoresis (SDS-PAGE). The separated proteins were transferred onto nitrocellulose membranes (0.2 μM pore size, Bio-Rad, Hercules, CA, USA) for 100 min at 70 volts using a Trans-blot SD semi-dry electrophoretic transfer cell (Bio-Rad). The transferred membranes were blocked with 0.5% BSA in Tris-buffered saline containing 0.1% Tween-20 (TBST) at room temperature (RT) for 2 h. After blocking, the membranes were washed three times with TBST for 10 min each with gentle shaking at RT. Next, the membranes were incubated with appropriate primary antibody (anti-MutS rabbit polyclonal; GeneCheck; #GC-M001 or anti-RecA antibody rabbit polyclonal; Abcam; #ab63797) at a 1:2000 dilution in TBST containing 0.5% BSA at 4 °C for 12 h with gentle shaking. Following three washes with TBST, the membranes were incubated with IRDye 800CW Goat anti-Rabbit IgG secondary antibody (LI-COR, #926-32211) at a 1:20,000 dilution in TBST containing 0.5% BSA at RT for 30 min with gentle shaking. After three washes with TBST, the protein bands were detected using the LI-COR Odyssey system. 

### 3.6. Cell Cultivations

10 mL cultures of medium (LB or M9G) and the appropriate antibiotic (ampicillin for the production of pUC19 vector or kanamycin for the production of pVAX vector) were grown in 50 mL Falcon tubes for 24 h (LB medium) and 36 h (M9G medium) at 37 °C with 250 rpm with an initial OD_600_ of 0.05. A 1 L shake flask culture containing 1 L of LB medium and kanamycin was grown in 3 L shake flasks for 24 h at 37 °C with 250 rpm with an initial OD_600_ of 0.05. In this study, we used a kanamycin concentration of 50 μg/mL and ampicillin concentration of 50 μg/mL. All the cultures were performed in triplicate.

### 3.7. Evaluation of Antibiotic Susceptibility by the Minimum Inhibitory Concentration Assay

The minimum inhibitory concentrations (MICs) of the selected antibiotics were measured against the tested strains (DH5α MG1655, and eMSD) following the general guidelines of the Clinical and Laboratory Standards Institute, with some modifications based on previous studies [34,35]. In brief, a single bacterial colony was cultured in LB broth until the mid-log phase (OD_600_ of 0.4–0.6). The bacterial suspension was then diluted with fresh LB to a concentration of 10^6^ colony-forming units (CFU)/mL. The number of CFU per mL was confirmed by plate count. Next, two-fold serial dilutions of antibiotics (from the working concentration of each antibiotics, 100 μg/mL of ampicillin, 25 μg/mL of chloramphenicol, 10 μg/mL of tetracycline, 50 μg/mL of kanamycin, and 100 μg/mL of carbenicillin) were prepared in fresh LB medium and mixed in a 1:1 ratio with the diluted bacterial culture, resulting in a final antibiotic concentration ranging from 0.78 to 50 μg/mL for ampicillin, 0.2 to 12.5 μg/mL for chloramphenicol, 0.08 to 5 μg/mL for tetracycline, 0.39 to 25 μg/mL for kanamycin, and 0.78 to 50 μg/mL for carbenicillin, with a final bacterial concentration of 5 × 10^5^ cells/mL. The mixtures were placed into a 96-well plate (SPL, #34096), with sterile LB as a negative control and LB inoculated with bacterial cells at a final concentration of 5 × 10^5^ cells/mL as growth control. The plates were placed in an incubator set at 37 °C with a rotation speed of 250 rpm. The MIC values were defined as the lowest concentrations of antibiotics at which no bacterial growth was observed at 600 nm using a microplate reader (Infinite 200 Pro; Tecan, Männedorf, Switzeland).

### 3.8. Statistical Analysis

The data are presented as mean ± standard deviation. All experiments were conducted in triplicate.

## 4. Conclusions

Genome-reduced *E. coli* has been extensively studied for protein and biochemical production, but its application in pDNA production has been understudied until now. In this study, we investigated the potential of the genome-reduced strain eMSD, generated by reintroducing the *mutS* gene into the eMS57 genome, for enhanced pDNA production. Our results demonstrated that eMSD has higher productivity of pUC19 and pVAX1 compared to the conventional strain DH5α, both at the small scale (10 mL culture) and the larger scale (1 L flask culture). Additionally, eMSD also showed increased sensitivity to various common antibiotic groups, which can be advantageous in biopharmaceutical production. These findings highlight the potential of the eMSD strain as a promising platform for efficient plasmid production in the biopharmaceutical industry. Moving forward, further engineering of this strain holds the potential for even greater improvements in both quantity and quality of pDNA production.

## Figures and Tables

**Table 1:** Plasmids and strains used in this study.

**Plasmids**	**Descriptions**	**Reference**
pUC19	pUC ori; Amp^R^	Enzynomics
pVAX1	pUC ori; Km^R^	Invitrogen
pACYCDuet-1	p15A ori; Cm^R^	Novagen
pCP22	FLP expression vector; Amp^R^	[32]
pREDI	Vector expresses λ-Red protein and I-SceI endonuclease; Amp^R^	[26]
pUC19-*nupG*	For construction of *mutS* DNA cassette; Amp^R^	This study
pUC19-*nupG*-FRT-CmR1	For construction of *mutS* DNA cassette; Amp^R^	This study
pUC19-*nupG*-FRT-*Cm^R^*-FRT-*spe*C	For construction of *mutS* DNA cassette; Amp^R^; Cm^R^	This study
pUC19-*nupG*-*mutS*-FRT-*Cm^R^*-FRT-*speC*	Vector contains *mutS* DNA cassette; Amp^R^; Cm^R^	This study
**Strains**	**Descriptions**	**Reference**
XL1-Blue		Stratagene
DH5α		Enzynomics
MG1655	*E. coli* K-12 strain	[33]
eMS57	Adaptive evolution strain in M9 glucose medium	[22]
eMS57*mutS^+^*	eMS57, *puuP::mutS-kan^R^*	[22]
eMSD	Reintroduction of *mutS* at the locus between *nupG* and *speC* in the eMS57 genome	This study

**Table 2:** Primers used in this study.

**Name**	**Sequence (5′-3′)**
XhoI_mutS_F	CGCTCGAGCCAACCGATACAATTTTGCGT
mutS_EcoRI_R	GCGAATTCGTTATTACACCAGGCTCTTCAAGC
nupG_out_F	GCCAACGCTTGGGTTAATCAACAC
speC_out_R	CCGCTGTTTGCTGCACTGGATG
XbanupGF	GACTCTAGAATCTGACCATCCCGTTCTTCTTAAG
nupGXENNR	GACCATATGCCATGGGAATTCCTCGAGCGGTAAAAAAAACGGGTCACCTTCTGGC
EcoRIFRTCmR1F	GTAACGAATTCGAAGTTCCTATTCTCTAGAAAGTATAGGAACTTCCTTTTGGCGAAAATGAGACGTTGATCGGC
CmR1NcoR	TGCCCATGGTGAAAACGGGGGCG
CmR2NcoF	GTTTTCACCATGGGCAAATATTATACGC
CmR2FRTR	GCGAGCCAACGGCGGCCCGCAAAAAAGAAGTTCCTATACTTTCTAGAGAATAGGAACTTCGGAATAGGAACTTCTTACGCCCCGCCCTGCCACTCATCG
oopspeCF	TTTTTTGCGGGCCGCCGTTGGCTCGCTTCTTACTTCAACACATAACCGTACAACC
SpeCNdeR	CACCATATGGTGTTGCCGAGCGTTTATAACAAG

## Data Availability

The datasets generated and/or analyzed during the current study are accessible from the corresponding author upon a reasonable request.
